# Effect of hypnotic communication on pain during arterial blood gas standardized procedures in the emergency department compared with traditional communication: a triple-blind randomized controlled trial (POPAIN study)

**DOI:** 10.1097/MEJ.0000000000001292

**Published:** 2025-11-21

**Authors:** Thomas Schmutz, Katia Iglesias, Franziska Peier, Vincent Ribordy, Viviane Donner, Jean-Luc Magnin, Youcef Guechi, Christophe Le Terrier

**Affiliations:** a Emergency, Ensemble Hospitalier de la Côte (EHC), Morges; b Department of Emergency Medicine, University of Fribourg and Fribourg Hospital; c School of Health Sciences, HES-SO University of Applied Sciences and Arts of Western Switzerland; d Division of Internal Medicine and Specialties, Department of Internal Medicine; e Central Laboratory, Fribourg Hospital, University of Fribourg and Fribourg Hospital, Fribourg; f Division of Intensive Care, Department of Acute Care Medicine, Geneva University Hospitals; g Department of Anesthesiology, Pharmacology, Intensive Care and Emergency Medicine, University of Geneva Faculty of Medicine, Geneva, Switzerland

**Keywords:** anxiety, arterial blood gas, emergency department, hypnosis, hypnotic communication, nocebo, pain, positive communication

## Abstract

**Background and importance:**

Although various strategies have been examined to mitigate discomfort during sampling, arterial blood gas (ABG) is a common and often painful procedure in emergency departments (EDs). Hypnotic communication, characterized by positive language/suggestions, may help reduce perceived procedural pain. Conversely, the traditional use of negative language may increase discomfort through a ‘nocebo’ effect.

**Objective:**

To assess whether hypnotic communication reduces procedural pain during ABG sampling compared with neutral or nocebo communication, when delivered by emergency physicians who have not received training in hypnosis.

**Design, setting, and participants:**

A single-center, triple-blind, randomized controlled trial with three parallel arms (hypnotic, neutral, and nocebo) was conducted from 4 April 2023 to 31 July 2024, in the ED of a Swiss Tertiary Care Hospital. All adult patients requiring ABG sampling were eligible for inclusion.

**Intervention:**

Three standardized communication scripts were used during a standardized procedure for ABG sampling: nocebo with negative words (e.g. ‘*I’m going to prick*’), neutral with neutral words (e.g. ‘*I am taking the sample*’), and hypnotic with positive words, and dissociative sentences (e.g. ‘*What is the noise of the lights at your home?’*). Communications were audio-recorded and independently reviewed to ensure protocol adherence.

**Outcomes measure and analysis:**

The primary outcome was the pain intensity, measured with a 0–10 numerical rating scale 3 min after the ABG sampling. Secondary outcomes included comfort and anxiety levels. Linear mixed-effects models were employed to conduct both intention-to-treat and per-protocol analyses.

**Main results:**

A total of 216 participants (median age 72 years; 57% male) were included (hypnotic, *n* = 71; nocebo, *n* = 71; neutral, *n* = 74). Dyspnea was the leading reason for ED consultation (*n* = 143; 66.2%). Hypnotic communication was associated with a statistically significant reduction in postprocedural pain compared with neutral communication [*β* = –0.97, 95% confidence interval (CI): –1.80 to 0.14, *P* = 0.02]; however, no significant differences were observed among the three groups in terms of median (interquartile range) pain scores [nocebo: 3 (1–5), neutral: 4 (2–6), hypnotic: 3 (1–5)], comfort or anxiety levels.

**Conclusion:**

Implementing hypnotic communication in the ED during ABG procedures did not lead to clinically meaningful reductions in pain, anxiety, or discomfort.

## Graphical abstract

**Figure:**
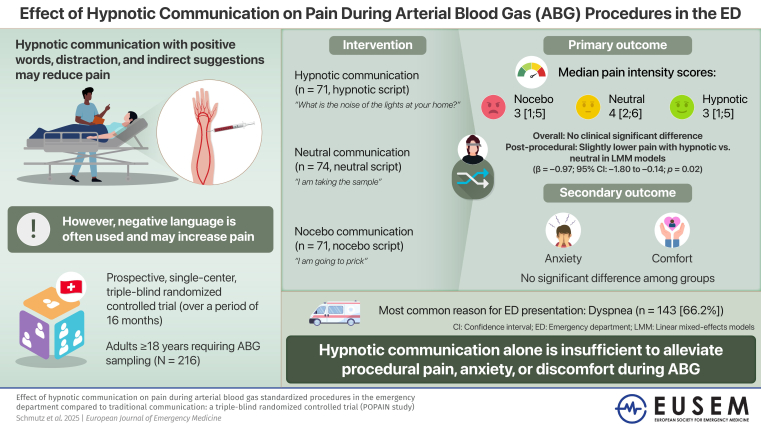

## Introduction

Acute pain is the most common symptom reported by patients admitted to the emergency department (ED) [1]. Despite the availability of numerous international guidelines, pain often remains undertreated [2–6]. In addition to pain associated with the presenting condition, procedural pain induced by a basic care intervention, such as blood sampling, has the potential to significantly contribute to patient discomfort [7]. Arterial blood gas (ABG) sampling is a routine, but painful, daily diagnostic procedure in the ED and remains essential for complex cases, mainly dyspnea, despite its increasingly limited indications [8]^9^,^10^]. Various strategies to reduce pain have been studied (e.g. cryotherapy, topical anesthetic creams, subcutaneous lidocaine injections, or the use of ultrasound) [11–13], but these are often impractical in ED settings because of time constraints and limited efficacy [14].

Beyond pharmacological interventions, communication has emerged as a simple, noninvasive tool to influence the perception of pain [15]. Hypnotic communication, using positive words, distraction, and indirect suggestions, has shown promise in modulating the pain experience [16–18]. By contrast, negative words can trigger a nocebo effect by amplifying the perception of pain. Warning patients about an impending painful procedure, although intended as empathetic communication, may paradoxically heighten pain [15,17,19,20].

Hypnotic communication has demonstrated benefits in some clinical contexts, notably in anesthesia and pediatric care [17,19,21]. Nevertheless, recent studies have reported inconsistent results regarding the independent effect of hypnotic communication on procedural pain in adults [22,23]. These findings underscore the context-dependence of these interventions [22,23]; however, no study has yet evaluated scripted hypnotic communication to reduce pain levels during blood sampling procedures in the ED in a rigorous triple-blind, randomized controlled design.

The objective of the POPAIN trial was to assess whether hypnotic communication could reduce patient self-reported procedural pain during ABG sampling in the ED compared with neutral or nocebo communication when delivered by emergency physicians without formal hypnosis training.

## Materials

### Study design and setting

This is a prospective, single-center center triple-blind, randomized controlled trial with three parallel arms aimed at evaluating the effect of hypnotic communication on procedural pain, anxiety, and comfort in patients undergoing ABG sampling compared with neutral communication or nocebo communication [24]. The study was conducted at the Fribourg Cantonal Hospital, Switzerland, an urban community teaching hospital with an annual ED census of over 40 000 visits. This study followed the principles of the Declaration of Helsinki and the CONSORT statement and was approved by the local ethics committee (CER-VD, BASEC 2022-00685) and registered at ClinicalTrials.gov (NCT05434169).

### Study enrollment

All adults (≥18 years) presenting to the ED between 4 April 2023 and 31 July 2024 who required ABG sampling as part of routine care were screened for eligibility. The indication for ABG sampling was determined by the treating physician independently of the study and according to local standard practice. When the indication for ABG was established, the treating physician contacted the triage physician who was responsible for verifying patient eligibility after obtaining verbal consent and ruling out any exclusion criteria (Table 1). All patients included provided written consent to participate in the study.

**Table 1:** Patient exclusion criteria

• Patients incapable of judgment • Patients with conditions that render them unable to participate in the intervention in this study (e.g. cognitive impairment, major hearing loss without the use of hearing aids, acute psychiatric disorders, under intravenous or oral sedation) • Patients with an insufficient understanding of the French language, as defined by self-evaluation • Patients in a critical situation requiring immediate resuscitation • Local anesthesia (subcutaneous, transdermal patch) at the point of ABG puncture • Patients already treated with anxiolytics or sedatives during care

### Randomization and blinding

Patients were randomly assigned in a 1 : 1 : 1 ratio to receive hypnotic, neutral, or nocebo communication using a computer-generated sequence with permuted blocks. Allocation concealment was ensured through sequentially numbered, opaque sealed envelopes prepared in advance by an independent administrator. Each envelope was linked to a unique patient identification code and securely stored on a password-protected server. The envelope contained the following items: (a) a digital voice recorder; (b) a pocket card specifying the exact sentences of the communication form to be used during the ABG; and (c) a section of the case report forms (CRFs) including the times and details of the procedural process to be completed by the treating physician performing the procedure. The treating physician was the only person allowed to see the contents of the envelope and, therefore the randomization, and they performed the intervention in a room closed off from the rest of the ED team. After the intervention, the triage physician was then called to enter in the room alone with the patient, to assess the outcome, unaware of the communication received. Data were collected using standardized paper CRFs before and after the procedure, supplemented by information extracted from the electronic medical record.

Patients were blinded to the specific objectives of the study and the randomization process. At the time of initial oral consent, participants received only partial oral information describing a study to assess the patient experience during ABG sampling with the aim to improve ED care, but without mentioning communication strategies or randomization. Patient blinding was lifted at the end of the procedure following outcome assessments. At that time, participants received full information about the study and signed the informed consent form. This two-step consent procedure was reviewed and approved by the local ethics committee. The research team analyzing the data were also blinded to study allocation. The term ‘triple-blind’ referred therefore to the patient, the outcome assessor (triage physician), and the analysis team.

### Intervention

The intervention consisted of implementing a standardized script communication strategy in addition to the routine procedure for ABG. Treating physicians had not received any formal training in hypnosis or hypnotic communication. They were instructed to perform ABG sampling according to one of three communication styles (hypnotic, neutral, and nocebo), delivered through standardized spoken scripts. Before each procedure, the treating physician opened the sealed envelope containing the pocket card in the examination room, which specified the exact sentences to be used during the procedure (Table 2). These sentences were adapted from previous studies [15,17]. The treating physician had to adhere to the assigned communication style during all steps of the procedure (site identification, skin disinfection, and ABG puncture). Each procedure was carried out in a private examination room with the door closed to minimize external visual and auditory distractions. The treating physician was alone in the room with the patient. In the event of a failed first attempt, a second puncture could be performed using a second pocket card with different phrases corresponding to the same communication strategy. After two unsuccessful attempts, the patient was excluded from the study. The entire procedure was recorded using a dedicated digital voice recorder.

**Table 2:** Description of the communication strategies used in each study arm

Communication strategy	Site identification	Skin decontamination	Puncture
Hypnotic (Structured positive verbal communication using distraction and indirect suggestion to modulate pain perception)	*‘Are you comfortably installed?’* Or *‘How old did you say you were?’*	*‘How did you get to the hospital?’* Or *‘What day is it?’*	*‘What is the noise of the lights at your home?’* Or *‘Does your car still go to the shops?’*
Neutral (Descriptive and factual language without emotional or suggestive connotation)	*‘I am taking your pulse’* Or *‘I am identifying the artery’*	*‘I am disinfecting the site’* Or *‘I am cleaning your skin’*	*‘I am taking the sample’* Or *‘I am performing the puncture’*
Nocebo (Common phrases used in ED with negative connotations, potentially enhancing pain perception)	*‘That’s where I am going to prick you’* Or *‘I am looking for the place where I will prick’*	*‘Watch out, it is cold’* Or *‘It is disagreeable’*	*‘Watch out, I am going to prick… 1, 2, 3…’* Or *‘This is going to hurt’*

ED, emergency department.

### Outcomes and measurements

The primary outcome was patient self-reported pain at 3 min postprocedure, assessed using a numeric rating scale (NRS) of 0 to 10 in which 0 represents no pain and 10 represents the worst imaginable pain [25,26]. NRS values at 3 min were compared to those obtained before the procedure.

Secondary outcomes included: patient self-reported perception of comfort and anxiety before and after the ABG procedure measured by a NRS scale of 0 to 10 for comfort (0 = no comfort and 10 = most comfortable imaginable), and anxiety (0 = no anxiety and 10 = worst anxiety imaginable) assessed at baseline (preprocedure) and 3 min postprocedure; global patient self-reported satisfaction with the communication strategy used, evaluated at the end of the procedure using a numeric scale of 0 to 10 (0 = extremely dissatisfied and 10 = extremely satisfied).

### Data collection

Data collection was performed jointly by the treating and triage physicians. The treating physician was responsible for completing the communication form and reporting procedural details (number of punctures and self-assessing their own stress level during the puncture on a numeric scale of 0 to 10). These data, together with the digital voice recording, were placed in the sealed envelope after the procedure and handed over to the triage physician. The triage physician completed standardized paper CRFs before and after the procedure. An independent data manager blinded to allocation entered data into a secure REDCap database.

Preprocedure data included patient demographics (age and sex), educational level, native language (French/Swiss German/German/Italian/Portuguese or other), tobacco use, diabetes, and vital signs. In addition, patients performed a self-assessment of pain, anxiety, and comfort using a 0–10 NRS. Data collected 3 min after the procedure included the following: vital signs; a second patient self-assessment of pain, anxiety, and comfort (reflecting values during the procedure); how the patient perceived the type of words used by the treating physician during the procedure (categorized as hypnotic, neutral, or negative); global patient satisfaction with the treating physician communication (recorded on a numeric scale of 0 to 10; 0 = extremely dissatisfied and 10 extremely satisfied); patient educational level; confirmation of the treating physician’s self-assessed stress level; and number of punctures performed.

All procedures were audio-recorded and independently reviewed to evaluate adherence to the assigned communication strategy. Two independent physicians not involved in patient care assessed all recordings to determine the communication strategy actually received by each patient. In cases of disagreement, a third physician adjudicated the recording to identify the most appropriate classification. Patients were (re)classified into one of three communication groups based on the presence of predefined keywords associated with each strategy (Supplementary Table S1, Supplemental digital content 1, https://links.lww.com/EJEM/A517). If at least one keyword from the nocebo list was identified, the patient was reassigned to the nocebo group.

### Sample size

Based on a group-independent *t* test, which represents a specific case of linear mixed-effects models (LMM), a sample size of 69 patients per group (totaling 207 participants) was calculated to demonstrate the intervention’s effectiveness on pain. This estimation was made with a significance level of 0.05, a statistical power of 0.90, a clinically meaningful difference of 1.5 points on the NRS for pain, and an SD of 2.5, applying the Bonferroni correction [17].

### Statistical analysis

Descriptive analysis (frequencies and percentages for categorical data, medians, and interquartile ranges for quantitative data) was produced for each time point, each group, and the overall study sample. Second, bivariate associations were computed to compare the groups at baseline (Pearson *χ*^2^ test, Fisher exact test, analysis of variances, and the Kruskal–Wallis test according to the distribution of variables). Third, to evaluate the effect of the intervention on the different outcomes, LMMs were performed, including physicians as random effects to account for clustering (25 physicians performed the procedure) and group, outcome at baseline, degree of emergency, and comorbidities, with age and gender as fixed effects. Models were tested using all available data. Intention-to-treat and per-protocol analyses were performed, including an analysis of effective communication received based on the communication attributed (after review by an independent physician). All statistical analyses were conducted using STATA software (version 14; StataCorp LLC, College Station, Texas, USA). The level of significance was set at *P* = 0.05 (two-tailed).

## Results

### Patient demographic characteristics

**Fig. 1:**
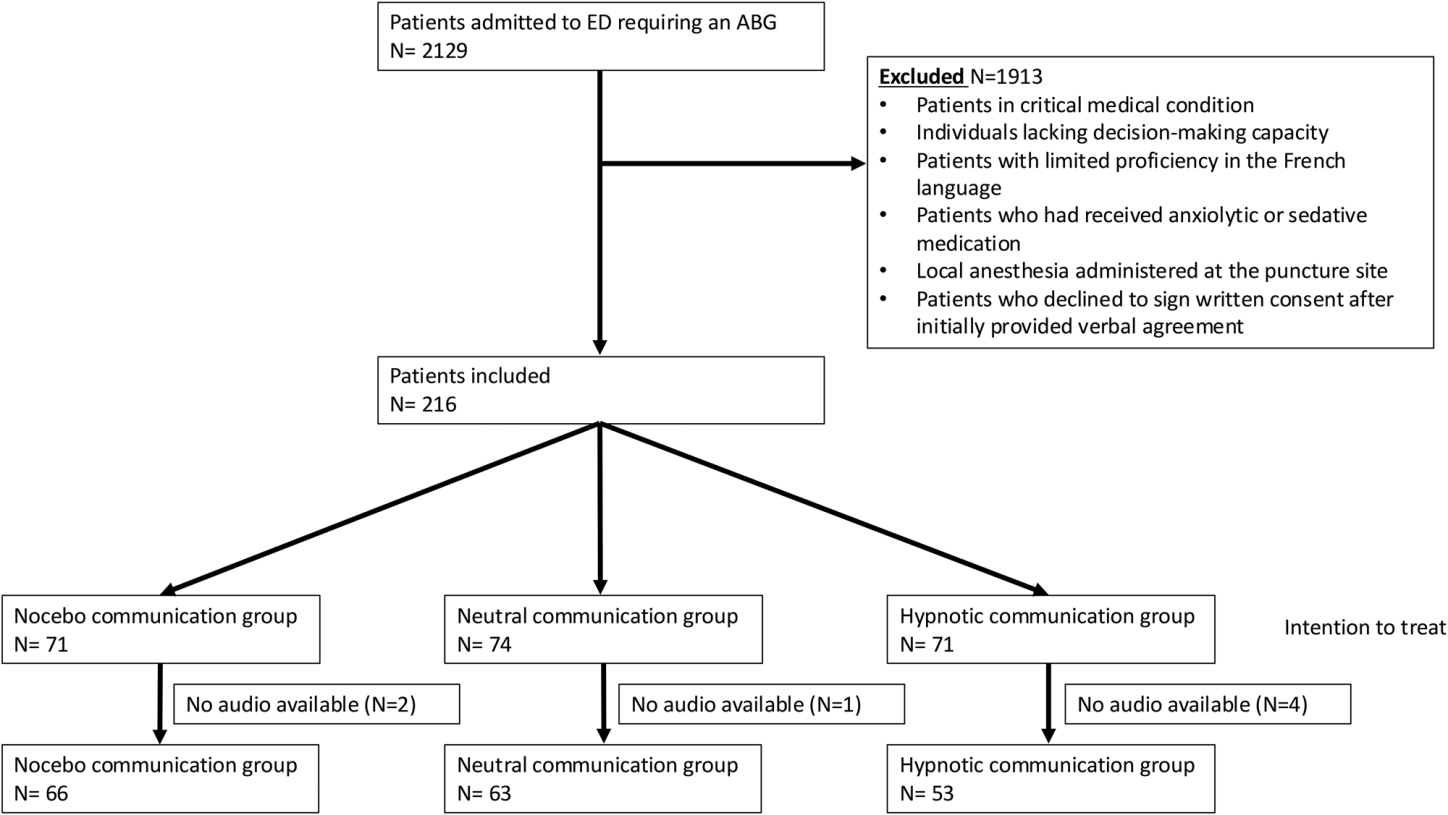
Study flow chart of patient inclusion and exclusion criteria. ED, emergency department.

Three participants were excluded at a later stage after initially providing verbal agreement but then declining to sign the written consent form. Therefore, a total of 216 patients were randomized into three groups: hypnotic (*n* = 71); neutral (*n* = 74); and nocebo (*n* = 71) (Fig. 1). Baseline demographic and clinical characteristics were well balanced across groups [median age (25–75%), 72 (59–81) years; male, 124 (57.4%); Table 3]. The most common reason for ED presentation was dyspnea [*n* = 143 (66.2%)]. Approximately 30% were active smokers, and 19% had diabetes. Patient severity was assigned as ‘emergency triage level 2’ for 55% of participants according to the Swiss Emergency Triage Scale. Median ED length of stay was 6.3 (4.9–9.1) h, and 82% were ultimately hospitalized. Although almost one-half of patients had a history of ABG [*n* = 95 (45%)] collection, only a few patients had experienced an ABG sampling within the last 24 h (7%). Around 19% (*n* = 42) of patients had received two punctures for ABG.

**Table 3:** Demographic characteristics of patients according to their randomization to communication strategies

Variable	All patients (*n* = 216)	Nocebo (*n* = 71)	Neutral (*n* = 74)	Hypnotic (*n* = 71)	*P* value
Age (years), median (25–75%)	72 (59–81)	75 (65–82)	72 (56–82)	70 (56–75)	0.033
Sex, *n* (%)					0.990
Men	124 (57%)	41 (58%)	42 (57%)	41 (58%)	
Women	92 (43%)	30 (42%)	32 (43%)	30 (42%)	
BMI (kg/m^2^), median (25–75%)	26 (23–29)	27 (5)	27 (6)	26 (6)	0.281
Comorbidities, *n* (%)					
Known diabetes	42 (19%)	14 (20%)	14 (19%)	14 (20%)	0.990
Active smokers	64 (30%)	19 (27%)	21 (28%)	24 (34%)	0.549
Past experienced of ABG	95 (44%)	26 (37%)	36 (49%)	33 (46%)	0.308
Education, *n* (%)					0.402^a^
Compulsory education	74 (34%)	27 (38%)	22 (30%)	25 (35%)	
Upper-secondary vocational education	88 (41%)	28 (39%)	33 (45%)	27 (38%)	
Tertiary education	48 (22%)	12 (17%)	19 (25%)	17 (24%)	
Unknown	6 (3%)	4 (6%)	0 (0%)	2 (3%)	
Emergency triage level (Swiss Emergency Triage Scale), *n* (%)					0.412
U1	56 (26%)	19 (27%)	14 (19%)	23 (32%)	
U2	118 (55%)	38 (54%)	46 (62%)	34 (48%)	
U3	42 (19%)	14 (20%)	14 (19%)	14 (20%)	
Reasons for ED admission, *n* (%)					0.314^a^
Dyspnea	143 (66%)	53 (75%)	48 (65%)	42 (59%)	
Chest pain	11 (5%)	3 (4%)	5 (7%)	3 (4%)	
Other	62 (29%)	15 (21%)	21 (28%)	26 (37%)	
Patient outcome after a stay in the emergency department, *n* (%)					0.140
Home	38 (18%)	8 (11%)	13 (18%)	17 (24%)	
Hospitalization	178 (82%)	63 (89%)	61 (82%)	54 (76%)	
Number of patients receiving two functions for ABG, *n* (%)	42 (19%)	16 (23%)	13 (18%)	13 (18%)	0.720
Before ABG, median (25–75%)					
Pain score	2 (0–4)	2 (0–4)	2 (0–4)	1 (0–4)	0.459
Anxiety score	3 (0–5)	3 (0–6)	3 (0–5)	3 (0–5)	0.759
Comfort score	7 (5–8)	7 (5–8)	7 (5–8)	7 (5–9)	0.901
Heart rate (pulse/min)	85 (73–97)	82 (73–98)	86 (70–96)	87 (75–101)	0.625
Respiratory rate (/min)	20 (16–25)	20 (16–27)	22 (15–26)	20 (16–24)	0.205
Systolic blood pressure (mmHg)	130 (116–149)	137 (118–154)	130 (117–147)	128 (115–150)	0.309
Diastolic blood pressure (mmHg)	75 (65–86)	76 (65–90)	74 (65–81)	78 (65–87)	0.219
Mean blood pressure (mmHg)	97 (85–111)	100 (84–114)	94 (88–107)	97 (85–112)	0.715
Time between ABG and outcome evaluation (min), median (25–75%)	5 (2–13)	7 (3–13)	5 (3–15)	5 (2–13)	0.496
After ABG, median (25–75%)					
Pain score	3 (1–5)	3 (1–5)	4 (2–6)	3 (1–5)	0.083^b^
Anxiety score	2 (0–4)	1 (0–4)	2 (0–4)	2 (0–4)	0.388
Comfort score	8 (5–9)	8 (5–9)	8 (5–9)	8 (6–10)	0.172
Heart rate (pulse/min)	84 (73–97)	83 (74–100)	88 (73–96)	84 (73–100)	0.790
Respiratory rate (/min)	20 (16–25)	20 (16–25)	20 (16–26)	18 (16–24)	0.238
Systolic blood pressure (mmHg)	131 (116–148)	132 (121–151)	124 (114–142)	132 (116–151)	0.242
Diastolic blood pressure (mmHg)	76 (67–87)	78 (66–88)	75 (67–88)	75 (66–83)	0.600
Mean blood pressure (mmHg)	98 (86–109)	99 (87–109)	99 (85–113)	93 (85–105)	0.242
Treating physician stress level, median (25–75%)	2 (1–3)	2 (1–3)	2 (1–3)	2 (1–3)	0.278
Patient satisfaction after procedure, median (25–75%)	9 (8–10)	9 (8–10)	9 (8–10)	10 (9–10)	0.081
Length of ED stay (h), median (25–75%)	6.3 (4.9–9.1)	7.4 (5.3–8.9)	7.4 (5.8–9.8)	6.3 (4.9–9.1)	0.175

ABG, arterial blood gas; ED, emergency department.

a *P*-values: For categorical variables, differences between groups were assessed using the *χ*^2^ test of independence, where Fisher’s exact test was used.

b For quantitative variables, differences between groups were assessed using the Kruskal–Wallis test, where one-way analysis of variance was used.

### Main results

No statistical differences were observed between groups regarding the vital parameters, including heart rate [85 (73–97) pulse/min], respiratory rate [20 (16–25)/min], systolic blood pressure [130 (116–149) mmHg], diastolic blood pressure [75 (65–86) mmHg] and mean blood pressure [97 (85–111), mmHg] (Table 3). The different outcomes were determined after a median of 5 (2–13) min after ABG sampling.

### Primary outcome: pain after arterial blood gas

**Fig. 2:**
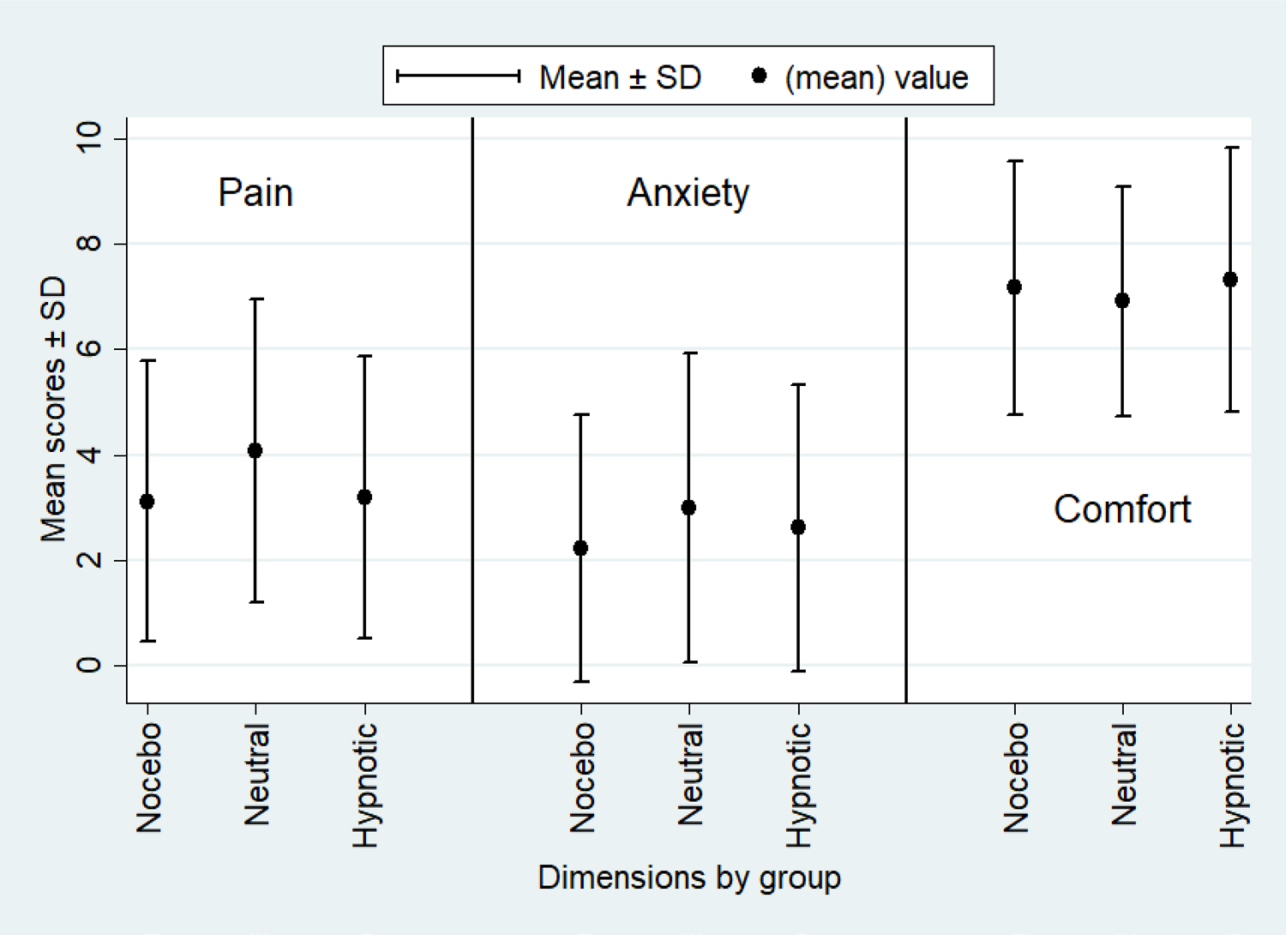
Mean pain scores assessed 3 min after arterial blood gas sampling according to each study arm (hypnotic, neutral, and nocebo groups).

Median pain scores assessed 3 min after ABG were as follows: hypnotic group 3 (1–5); neutral group 4 (2–6); and nocebo group 3 (1–5) (Table 3 and Fig. 2). The median pain intensity was relatively low across all groups, being at 3 (1–5).

In the intention-to-treat analysis, LMMs, with treating physician as a random effect, showed that hypnotic communication was associated with a statistically significant reduction in postprocedural pain compared with neutral communication [*β* = –0.97, 95% confidence interval (CI): –1.80 to –0.14, *P* = 0.02], even if no significant clinical differences were observed between the groups as reported in Table 4. No statistically significant difference was observed between the nocebo and neutral groups (*β* = –0.09, 95% CI: –0.91 to 0.74, *P* = 0.83).

**Table 4:** Intention-to-treat analyses of the impact of the communication strategy on pain, anxiety, and comfort (after arterial blood gas sampling): linear mixed-effects models with treating physician as a random effect

Variable	Outcomes								
	Pain			Anxiety			Comfort		
	Coef	95% CI	*P* value	Coef	95% CI	*P* value	Coef	95% CI	*P* value
Respective outcome before ABG	0.31	(0.18–0.45)	<0.001	0.33	(0.21–0.44)	<0.001	0.51	(0.40–0.62)	<0.001
Age	‐0.01	(‐0.04 to 0.01)	<0.001	‐0.002	(‐0.02 to 0.02)	0.81	0.007	(‐0.01 to 0.02)	0.41
Gender									
Male	0.11	(‐0.57 to 0.80)	0.75	‐0.30	(‐0.97 to 0.37)	0.38	0.19	(‐0.37 to 0.75)	0.50
Past experience of ABG	0.44	(‐0.28 to 1.16)	0.24	0.06	(‐0.97 to 0.37)	0.87	‐0.16	(‐0.74 to 0.42)	0.58
Emergency triage level									
U1	‐0.10	(‐0.92 to 0.72)	0.81	‐0.08	(‐0.88 to 0.73)	0.85	0.74	(0.07–1.41)	0.03
U3	0.34	(‐0.57 to 1.25)	0.46	‐0.26	(‐1.14 to 0.62)	0.56	0.82	(0.08–1.55)	0.03
Groups (reference = neutral)									
Nocebo	‐0.68	(‐1.50 to 0.15)	0.11	‐0.79	(‐1.60 to ‐0.02)	0.06	0.13	(‐0.55 to 0.81)	0.71
Hypnotic	‐0.97	(‐1.8 to ‐0.14)	0.02	‐0.58	(‐1.39 to ‐0.22)	0.2	0.32	(‐0.35 to 1.00)	0.35

ABG, arterial blood gas; Coef, coefficient; DBP, diastolic blood pressure; ED, emergency department; MBP, mean blood pressure; SBP, systolic blood pressure.

### Secondary outcomes: anxiety, comfort, and satisfaction

Median anxiety scores decreased from 3 (0–5) before ABG to 2 (0–4) post-ABG.

Although the reduction in anxiety in the hypnotic group did not reach statistical significance, it exhibited a favorable trend (*β* = –0.58, 95% CI: –1.39 to –0.22, *P* = 0.20). The nocebo strategy demonstrated no significant reduction in anxiety (*β* = –0.79, 95% CI: –1.60 to –0.02, *P* = 0.06). There was a slight improvement in comfort scores across all groups (from 6.7 ± 2.5 to 7.2 ± 2.4), with no significant differences observed between the study arms. Overall, no statistically significant between-group differences were identified in the adjusted models (*P* > 0.05 for all comparisons). Interestingly, patient satisfaction with the communication strategy was high overall, with a median score of 9 (8–10).

Per-protocol analysis showed similar characteristics and results for all outcomes, as shown in Supplementary Figure S1, Tables S2, and S3, Supplemental digital content 1, https://links.lww.com/EJEM/A517. Additional analysis in patients who experienced first-time ABG sampling did show similar results for all outcomes in both intention-to-treat and per-protocol analyses (Supplementary Tables S4 and S5, Supplemental digital content 1, https://links.lww.com/EJEM/A517). Interestingly, in this subgroup, hypnotic communication was associated with a statistically significant reduction in postprocedural pain compared with neutral communication [*β* = –1.59, 95% CI: –2.99 to –0.19, *P* = 0.03] in per-protocol analysis, as shown in Supplementary Table S5, Supplemental digital content 1, https://links.lww.com/EJEM/A517.

### Predictors of procedural outcomes

Baseline pain, anxiety, and comfort scores were the strongest predictors of postprocedural ratings (*β* range: 0.31–0.51, *P* < 0.001). Age, sex, prior ABG experience, and treating physician stress levels were not significantly associated with pain or anxiety scores.

## Discussion

Overall, in contrast to our initial hypothesis, hypnotic communication did not result in a clinically significant reduction in patient self-reported pain, anxiety, or discomfort. These results contrast with studies conducted in more controlled environments, such as operating rooms, where hypnotic communication has demonstrated consistent reductions in pain and anxiety [17]. Several contextual factors may explain the limited clinical effect observed. First, the relatively low average pain intensity after ABG likely introduced a floor effect, limiting room for improvement, an issue also reported in recent ED trials using communication-based strategies [17,23]. Second, the ED setting may not be optimal for understanding suggestive language because of environmental stressors such as noise, urgency, and cognitive overload. Third, the predominance of older dyspneic patients may have reduced receptiveness to verbal modulation as cognitive availability and attention are critical for suggestibility when compared with the KTHYPE study population (median age: 72 vs. 54 years) [17,27]. Cognitive impairment has been documented as a factor that impedes an individual’s capacity to self-report pain. To date, no specific pain-assessment tool has been recognized as a gold standard within this particular context [27,28]. The specific characteristics of our cohort (older age, predominantly dyspneic presentations) also further limit the generalizability of our results to younger or more procedure-naive ED populations, in whom the impact of hypnotic communication may differ. Nevertheless, our findings appear to be in alignment with recently published results in other settings, such as intensive care units, where patients encounter similar environmental challenges [29].

Finally, the intervention focused solely on verbal scripting in physicians who were not trained in hypnosis, although the literature increasingly highlights the essential role of nonverbal communication (intonation, posture, and pacing) in achieving meaningful hypnotic effects [30]. This is consistent with previous research, which also failed to demonstrate a significant benefit of hypnotic verbal communication on pain during peripheral intravenous catheter placement in the ED [23]. Taken together, these results underscore the contextual complexity of applying communication-based interventions in acute care and suggest that verbal scripts alone may be insufficient without accompanying relational or environmental adjustments. Future research should consider combining verbal and nonverbal communication strategies, assessing both subjective and objective outcomes, and evaluating the impact of provider training in hypnotic communication. Targeting more receptive subgroups, such as patients with high procedural anxiety or no prior exposure to ABG, may also enhance the effectiveness of such interventions.

### Limitations

Although designed with methodological rigor, the study was conducted at a single center, which may limit the generalizability of the findings. Second, the study included only junior medical practitioners without formal training in hypnosis or therapeutic communication, as these individuals typically perform ABG in real-life practice. Although physicians delivered standardized scripts, nonverbal elements (e.g. tone, empathy, posture, and pacing) were neither standardized nor measured. Differences between operators were incorporated in the mixed-effects model as a random factor, but this statistical approach does not identify the specific sources of variability, and could not be explored further because of the low number of procedures per operator. Therefore, potential bias related to unmeasured nonverbal communication cannot be excluded [31,32]. In addition, about half of the participants had a history of ABG sampling, but the study did not differentiate between positive and negative prior experiences, which may have affected pain perception. Third, organizational constraints (patient flow, ED overcrowding, and task interruptions) occasionally delayed outcome assessment, introducing a potential recall bias. Pain was sometimes assessed several minutes after the procedure, possibly blunting the memory of peak pain. Nevertheless, no discrepancies in time assessment were identified among the three groups. Moreover, the stringent adherence criteria employed to ensure methodological rigor resulted in a key limitation of the study: a relatively lower adherence rate of 79% in the hypnotic group. This is because strict adherence to standardized verbal scripts is challenging in real-world ED settings. Finally, ABG was selected for this study as a routine and relatively simple procedure to represent a pain-inducing experience in the ED. Although hypnotic communication was statistically associated with a reduction in postprocedural pain compared with neutral communication, especially among patients undergoing ABG sampling for the first time, many participants reported low levels of pain. This may have limited the study’s ability to detect differences between communication strategies. Future studies should evaluate the impact of hypnotic communication and nonverbal components on pain relief during more painful procedures, where its effect may be more pronounced and clinically relevant.

### Conclusion

This study did not observe any clinically significant benefit of hypnotic communication on levels of pain, anxiety, and comfort when performing ABG in an ED. This highlights the fact that communication alone is insufficient to influence the pain experienced by patients in the emergency context. The nonverbal attitude and communication training of the operators, not evaluated in this work, combined with the mode of communication, could play a major role.

## Acknowledgements

We would like to thank the entire emergency team for their help in conducting this study alongside their clinical work.

We also thank Rosemary Sudan for her help in the final editorial assistance.

This work was funded by a grant from Fribourg Hospital (Grant-2019). The funder had no role in the study design, data collection, data analysis, data interpretation, or writing of the report.

T.S. conceptualized the study, while T.S., C.L.T., K.I., F.P., and Y.G. participated in the study’s design and data acquisition. K.I. provided statistical advice on the study’s design and participated in the data analysis. The subsequent analysis of the data were conducted by T.S. and C.L.T. T.S. was responsible for drafting the initial version of the manuscript and served as the principal investigator. The supervision of the trial was overseen by C.L.T., who also reviewed the initial draft and served as the senior author. T.S. and VR obtained funding. All authors made significant contributions to the revision of the manuscript and approved the final version.

The results of this study were presented at the annual meeting of the French Society of Emergency Medicine in Paris, France, 4–6 June 2025.

The data underlying this article may be shared upon reasonable request to the corresponding author and in accordance with Swiss legislation and applicable data protection regulations, and if approved by the relevant local ethics committee.

### Conflicts of interest

There are no conflicts of interest.

## Supplementary Material


