# Supplementary material for: Effect of hypnotic communication on pain during arterial blood gas standardized procedures in the emergency department compared with traditional communication: a triple-blind randomized controlled trial (POPAIN study)
Source: Eur J Emerg Med. 2025 Nov 21;33(3):185–93. doi: 10.1097/MEJ.0000000000001292 (PMC13124270; doi:10.1097/MEJ.0000000000001292)
Supplement: Supplementary file 1 [file ejem-33-185-s001.pdf]

Table S1. Group assignment keywords.

**Nocebo Group**	**Hypnotic Group**
Pain	Be reassured
Cold	Are you comfortable ?
Prick	Are you relieved
Do not panic	I am going to place the drip
Are you in pain ?	Have confidence
This is going to sting	Take it easy
Do not move, it won’t be long	It will be fast
Do not be frightened	Do you feel well ?
How do you feel ?	
I am going to prick	

Table S2. Demographic characteristics of patients included in the per-protocol analysis.

**Variable**	**All patients (*n*=182)**	**Nocebo (*n*=66)**	**Neutral (n=63)**	**Hypnotic (*n*=53)**	**p-value**
Age (years), median [25%;75%]	72 [59;82]	75 [65;82]	72 [53;83]	68 [57;75]	0.090
Sex, *n* (%)					0.757
Men	101 (55 %)	39 (59 %)	34 (53 %)	28 (54 %)	
Women	81 (45 %)	27 (41 %)	29 (47 %)	25 (46 %)	
Body mass index (kg/m^2), median [25%;75%]	25.7 [23.0;29.1]	26.4 [23.7;29.1]	25.9 [22.8;30.1]	24.7 [22.2;28.3]	0.279
Comorbidities, *n* (%)					
Known diabetes	33 (18 %)	13 (20 %)	11 (17 %)	9 (17 %)	0.916
Active smokers	54 (30 %)	18 (27 %)	17 (27 %)	19 (37 %)	0.457
Past experienced of ABG	80 (44 %)	25 (38 %)	29 (46 %)	26 (51 %)	0.350
Education, *n* (%)					0.600*
Compulsory education	65 (36 %)	24 (36 %)	19 (30 %)	22 (42 %)	
Upper-secondary vocational education	76 (42 %)	26 (39 %)	30 (48 %)	20 (38 %)	
Tertiary education	36 (20 %)	12 (18 %)	14 (22 %)	10 (19 %)	
Unknown	5 (3 %)	4 (6 %)	0 (0 %)	1 (2 %)	
Emergency triage level (Swiss Emergency Triage Scale), *n* (%)					0.452
U1	46 (25 %)	17 (26 %)	12 (19 %)	17 (32 %)	
U2	100 (55 %)	35 (53 %)	40 (63 %)	25 (47 %)	
U3	36 (20 %)	14 (21 %)	11 (17 %)	11 (21 %)	
Reasons for ED admission, *n* (%)					0.281*
Dyspnea	121 (66 %)	48 (73 %)	41 (65 %)	32 (60 %)	
Chest pain	10 (5 %)	3 (5 %)	5 (8 %)	2 (4 %)	
Other	51 (29 %)	15 (22 %)	17 (27 %)	19 (36 %)	
Patient outcome after a stay in the emergency department, *n* (%)					0.297
Home	29 (16 %)	7 (11 %)	11 (17 %)	11 (21 %)	
Hospitalization	153 (84 %)	59 (89 %)	52 (83 %)	42 (79 %)	

Number of patients receiving 2 punctions for ABG, *n* (%)	30 (16 %)	15 (23 %)	9 (14 %)	6 (11 %)	0.211
Before ABG, median [25%;75%]					
Pain score	2 [0;4]	2 [0;4]	2 [0;5]	2 [0;4]	0.602
Anxiety score	3 [0;5]	3 [0;6]	3 [0;5]	4 [1;5]	0.812
Comfort score	7 [5;8]	8 [5;8]	7 [6;8]	7 [5;9]	0.972
Heart rate (pulse/min)	84 [73;96]	82 [73;95]	88 [75;95]	85 [72;100]	0.939
Respiratory rate (/min)	20 [15;25]	20 [16;26]	22 [15;26]	20 [16;24]	0.715
Systolic blood pressure (mmHg)	130 [116;148]	136 [118;153]	130 [114;147]	128 [115;147]	0.244
Diastolic blood pressure (mmHg)	75 [66;85]	77 [65;89]	73 [63;81]	79 [67;84]	0.135
Mean blood pressure (mmHg)	98 [87;110]	100 [85;114]	94 [87;107]	97 [87;111]	0.514
Time between ABG and outcome evaluation (minutes), median [25%;75%]	6 [3;13]	6 [3;11]	5 [3;15]	5 [2;13]	0.525
After ABG, median [25%;75%]					
Pain score,	3 [1;5]	3 [1;5]	4 [2;6]	4 [0;5]	0.097**
Anxiety score,	2 [0;5]	2 [0;4]	2 [0;5]	2 [0;5]	0.366
Comfort score,	8 [5;9]	8 [5;9]	7 [5;9]	8 [6;10]	0.408
Heart rate (pulse/min)	84 [73;96]	82 [73;95]	88 [75;95]	85 [72;100]	0.880
Respiratory rate (/min)	20 [16;25]	20 [15;26]	20 [16;26]	19 [15;24]	0.430
Systolic blood pressure (mmHg)	131 [116;147]	132 [121;151]	128 [114;142]	132 [116;148]	0.254
Diastolic blood pressure (mmHg)	76 [67;84]	78 [67;87]	75 [66;83]	75 [69;84]	0.423
Mean blood pressure (mmHg)	98 [87;109]	99 [89;109]	96 [85;106]	99 [88;112]	0.276
Treating Physician stress level, median [25%;75%]	2 [1;3]	2 [1;3]	2 [0;3]	1 [0;2]	0.080
Patient satisfaction after procedure, median [25%;75%]	9 [8;10]	9 [8;10]	9 [8;10]	10 [8;10]	0.116
Length of ED stay (hours), median [25%;75%]	6.9 [5.2;9.0]	6.9 [5.2;8.7]	7.3 [5.8;9.9]	6.1 [4.4;8.7]	0.043

Abbreviations: SD: Standard deviation; ABG, arterial blood gas; ED, emergency department; P-values: For categorical variables, differences between groups were assessed using the chi-squared test of independence, unless marked with *, where Fisher's exact test was used. For quantitative variables, differences between groups were assessed using the Kruskal–Wallis test, unless marked with **, where one-way ANOVA was used.

Figure S1. Mean pain scores assessed 3 min after arterial blood gas sampling according to each study arm (hypnotic, neutral and nocebo groups) of patients included in the per-protocol analysis.

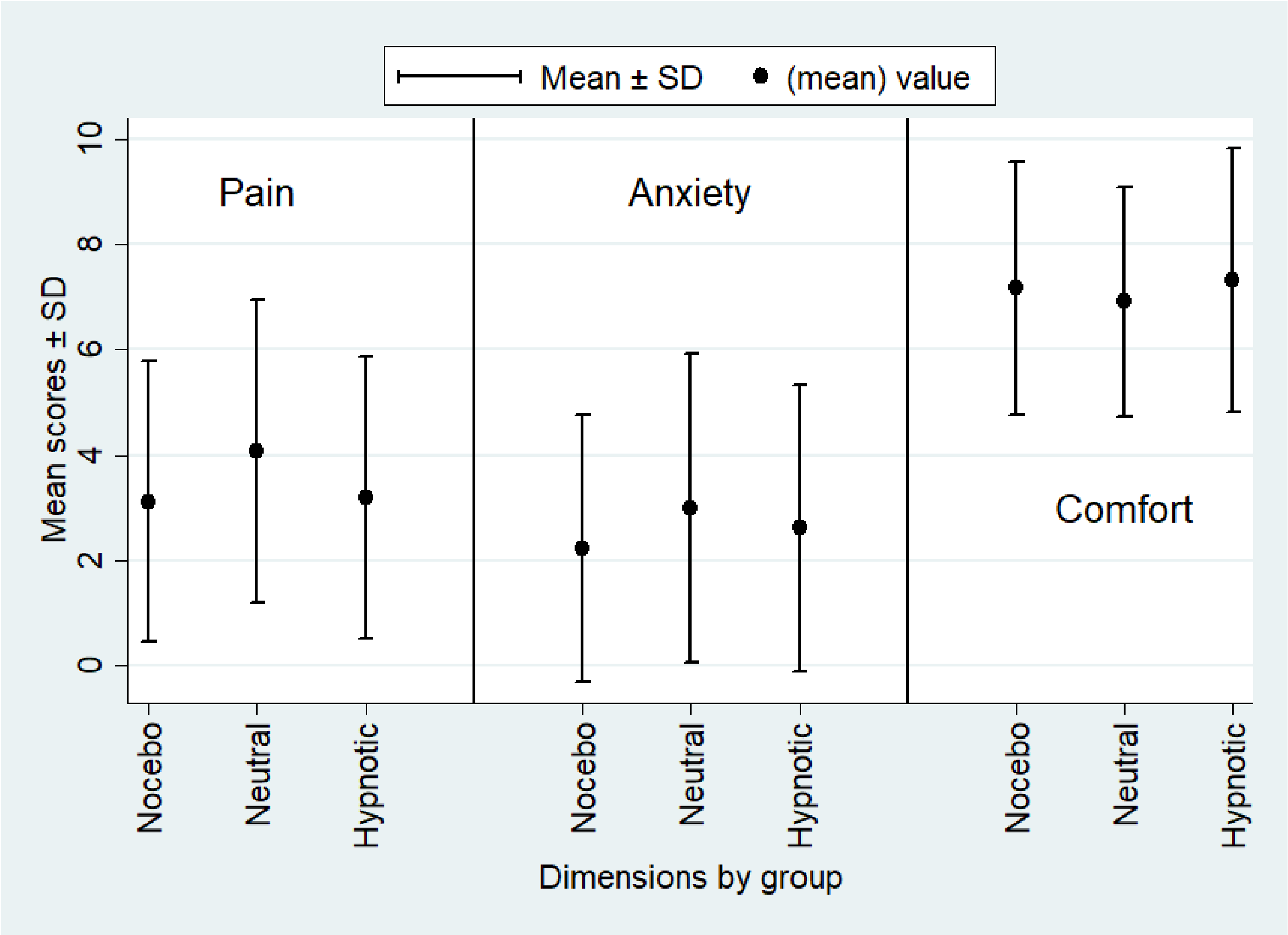

Table S3. Per-protocol analyses of the impact of the communication strategy on pain, anxiety and comfort (after arterial blood gas sampling): linear mixed-effects models with treating physician as a random effect.

	Outcomes								
	**Pain**			**Anxiety**			**Comfort**		
Variable	Coef	95% CI	*P* value	Coef	95% CI	*P* value	Coef	95% CI	*P* value
Respective outcome before ABG	0.33	[0.19; 0.47]	<0.001	0.33	[0.20; 0.45]	<0.001	0.57	[0.45; 0.69]	<0.001
Age	-0.02	[-0.04; 0.01]	0.12	-0.003	[-0.03; 0.02]	0.78	0.007	[-0.01; 0.02]	0.41
Gender									
Male	0.70	[-0.68; 0.82]	0.86	-0.14	[-0.89; 0.62]	0.72	0.20	[-0.36; 0.77]	0.48
Past experienced of ABG	0.29	[-0.51; 1.09]	0.47	0.08	[-0.85; 0.70]	0.85	-0.05	[-0.65; 0.54]	0.86
Emergency triage degree									
U1	-0.10	[-1.01; 0.80]	0.82	-0.12	[-1.04; 0.80]	0.80	0.61	[-0.08; 1.31]	0.08
U3	0.29	[-0.69; 1.28]	0.56	-0.34	[-1.32; 0.65]	0.50	0.95	[0.21; 1.70]	0.01
**Groups (reference=neutral)**									
Nocebo	-0.74	[-1.62; 0.14]	0.10	-0.87	[-1.76; 0.13]	0.05	0.14	[-0.36; 0.77]	0.48
Hypnotic	-1.03	[-1.98; -0.82]	0.03	-0.59	[-1.53; 0.34]	0.22	0.28	[-0.65; 0.54]	0.86

Abbreviations: Coef, Coefficient; ABG, arterial blood gas; ED, emergency department; BMI, body mass index; SBP, systolic blood pressure; DBP, diastolic blood pressure; MBP, mean blood pressure.

Table S4. Intention-to-treat subgroup analyses of the impact of the communication strategy on pain, anxiety and comfort (after arterial blood gas sampling) in patient who experienced first-time of ABG sampling: linear mixed-effects models with treating physician as a random effect.

	**Outcomes**								
	**Pain**			**Anxiety**			**Comfort**		
Variable	Coef	95% CI	*P* value	Coef	95% CI	*P* value	Coef	95% CI	*P* value
Respective outcome before ABG	0.21	[0.14; 0.40]	0.04	0.26	[0.11; 0.41]	0.001	0.45	[0.30; 0.61]	<0.001
Age	-0.005	[-0.03; 0.02]	0.73	-0.01	[-0.02; 0.04]	0.45	0.02	[-0.05; 0.04]	0.13
Gender									
Male	0.62	[-0.32; 1.55]	0.20	-0.76	[-1.68; 0.17]	0.11	0.33	[-0.38; 1.03]	0.36
Emergency triage degree									
U1	-0.75	[-2.05; 0.55]	0.26	-0.53	[-1.82; 0.77]	0.43	0.73	[-0.26; 1.71]	0.15
U3	-0.12	[-1.21; 0.97]	0.83	-0.21	[-1.28; 0.87]	0.70	0.32	[-0.51; 1.15]	0.45
**Groups (reference=neutral)**									
Nocebo	-0.47	[-1.56; 0.62]	0.84	-0.93	[-2.05; 0.18]	0.11	0.73	[-0.12; 1.57]	0.09
Hypnotic	-1.27	[-2.50; -0.49]	0.04	-0.33	[-1.52; 0.87]	0.59	1.00	[-0.08; 1.92]	0.03

Abbreviations: Coef, Coefficient; ABG, arterial blood gas; ED, emergency department; BMI, body mass index; SBP, systolic blood pressure; DBP, diastolic blood pressure; MBP, mean blood pressure.

Table S5. Per-protocol subgroup analyses of the impact of the communication strategy on pain, anxiety and comfort (after arterial blood gas sampling) in patient who experienced first-time of ABG sampling: linear mixed-effects models with treating physician as a random effect.

	**Outcomes**								
	**Pain**			**Anxiety**			**Comfort**		
Variable	Coef	95% CI	*P* value	Coef	95% CI	*P* value	Coef	95% CI	*P* value
Respective outcome before ABG	0.22	[0.02; 0.42]	0.03	0.25	[0.08; 0.43]	0.005	0.54	[0.39; 0.70]	<0.001
Age	-0.006	[-0.04; 0.02]	0.68	-0.01	[-0.02; 0.04]	0.45	0.01	[-0.08; 0.03]	0.27
Gender									
Male	0.78	[-0.20; 1.76]	0.12	-0.69	[-1.72; 0.34]	0.19	0.36	[-0.31; 1.03]	0.30
Emergency triage degree									
U1	-0.73	[-2.20; 0.75]	0.34	-0.66	[-2.17; 0.86]	0.40	0.59	[-0.43; 1.60]	0.26
U3	-0.34	[-1.51; 0.82]	0.56	-0.38	[-1.58; 0.83]	0.54	0.63	[-0.18; 1.43]	0.13
**Groups (reference=neutral)**									
Nocebo	-0.69	[-1.83; 0.45]	0.23	-0.97	[-2.17; 0.24]	0.12	0.87	[0.08; 1.66]	0.03
Hypnotic	-1.59	[-2.99; -0.19]	0.03	-0.31	[-1.71; 1.09]	0.67	1.26	[0.30; 2.21]	0.01

Abbreviations: Coef, Coefficient; ABG, arterial blood gas; ED, emergency department; BMI, body mass index; SBP, systolic blood pressure; DBP, diastolic blood pressure; MBP, mean blood pressure.
